# Green extraction of phenolics and flavonoids from black mulberry fruit using natural deep eutectic solvents: optimization and surface morphology

**DOI:** 10.1186/s13065-023-01041-x

**Published:** 2023-09-21

**Authors:** Tan Phat Vo, Thuy Vy Pham, Kasia Weina, Thi Ngoc Huyen Tran, Le Thao Vy Vo, Phuc Thanh Nguyen, Tran Linh Ha Bui, Thuy Han Phan, Dinh Quan Nguyen

**Affiliations:** 1 Laboratory of Biofuel and Biomass Research, Faculty of Chemical Engineering, Ho Chi Minh City University of Technology (HCMUT), 268 Ly Thuong Kiet Street, District 10, Ho Chi Minh City, Vietnam; 2 Vietnam National University Ho Chi Minh City, Linh Trung Ward, Thu Duc City, Ho Chi Minh City, Vietnam; 3 Evergreen Labs, My Khue Ward, Danang, Vietnam

**Keywords:** Natural deep eutectic solvents, Ultrasonic-assisted extraction, Optimization, Phenolics, Black mulberry fruit, Flavonoids

## Abstract

This study deployed ultrasonic-assisted extraction (UAE), combined with natural deep eutectic solvents (NADES), to extract phenolics and flavonoids from the black mulberry fruit, and the antioxidant activity was examined. The extraction yields of NADES-based UAE were assessed based on the yields of phenolics and flavonoids extracted from the black mulberry fruit. This study selected the molar ratios of hydrogen bond acceptors (HBA) and hydrogen bond donors HBD at 1:2 from previous studies. Choline chloride-lactic acid showed the highest solubility with phenolics and flavonoids among NADES systems. One-factor experiments evaluated the effect of UAE conditions (liquid-to-solid ratio (LSR), water content in NADES, temperature, and time) on TPC, TFC, and antioxidant activity. The suitable NADES-based UAE conditions for extracting phenolics and flavonoids from the black mulberry fruit were 60 ml/g of LSR, 40% water content, 70 °C, and 15 min. Response surface methodology with the Box-Behnken design model optimized the NADES-based UAE process based on response (TPC, TFC, ABTS, OH, and DPPH). The optimal conditions for the NADES-based UAE process were 70 ml/g of LSR, 38.9% water content in NADES, 67.9 °C, and 24.2 min of extraction time. The predicted values of the Box-Behnken design were compatible with the experimental results. Moreover, scanning electron microscopy (SEM) was used to survey the surface of black mulberry fruit with and without sonication. SEM can assist in demonstrating the destructive effect of NADES and ultrasonic waves on material surfaces. SEM findings indicated the high surface destruction capacity of NADES, which partially contributed to a superior extraction yield of NADES than conventional organic solvents. The study proposes an efficient and green method for extracting bioactive compounds from black mulberry fruits. The black mulberry fruit extracts can be applied to meat preservation and beverages with high antioxidants.

## Introduction

Black mulberry (*Morus nigra L.)* is one of the most common members of the genus *Morus* of the family *Moraceae,* along with white mulberry (*Morus alba L*.) and red mulberry (*Morus rubra L*.) [1]. Mulberry is widely distributed among subregions of Asia, North America, and Africa; their leaves are usually used as nutrient sources for silkworms (*Bombyx mori L.)* in Asia countries [2, 3]. Mulberry fruits can be an ingredient in several food products, such as jams, ice creams, vinegar, juices, and wine [4]. Black mulberry fruits possess a high content of bioactive components such as phenolic compounds, flavonoids, anthocyanins, and organic acids that benefit human health [5]. Phenolic acids (gallic, syringic, caffeic, and neo-chlorogenic acids) and flavonoids (rutin and quercetin) occur naturally in black mulberry fruit. These compounds have various nutraceutical functions, including antioxidant, anti-microbial, anti-hyperglycemic, anti-hyperlipidemic, anti-inflammatory, anti-cancer, and neurodegenerative properties [5, 6]. Phenolic and flavonoid compounds quench reactive oxygen species (ROS), triggering lipid oxidation, denaturing protein, and breaking DNA chains [7–9]. Natural compounds protect the skin and organs from the external environment because of these functions [10, 11]. The multitude of functions these bioactive compounds have on human health highlights the need to evaluate a reliable extraction method.

Extraction is an analytical technique to separate desired compounds from a solid matrix. Some drawbacks of traditional extraction include a large amount of solvent required and a prolonged extraction time. These challenges are difficult to overcome while maintaining an effective extraction process. Several methods have been developed to minimize the bottlenecks in conventional techniques, such as microwave-assisted extraction (MAE) and supercritical fluid extraction (SFE) [12]. However, MAE requires a significant amount of energy consumption, while SFE requires expensive devices [13]. Ultrasound-assisted extraction (UAE) is based on the formation, growth, and implosion of cavitation bubbles in the cyclical pattern of rarefaction and compression, leading to the generation of extreme temperature and pressure in the microenvironment [14]. These phenomena can facilitate cell wall disruption and mass transfer due to the generation of high shearing forces in the extraction medium [15]. UAE can be considered as an efficient, safe, and green technique since it can be conducted at lower temperatures, which is suitable for thermolabile compounds and minimum structural alternation when using an ultrasonic bath. UAE also shortens extraction time and reduces solvent usage and running costs [12].

The concept of deep eutectic solvents (DES) has been widely adopted due to the discovery of Abbott et al. [16]. NADES is a kind of DES composed of organic compounds, such as sugars, organic acids, and bases in living cells [17]. NADES is a combination of a hydrogen-bond acceptor (HBA) and hydrogen-bond donor (HBD) with a low melting point [18]. NADES can be regarded as a class of ionic liquids with similar physicochemical properties, such as high chemical and thermal stability, non-flammability, low volatility, recyclability, high tunability, and high solubilization capacity for various compounds [19]. Moreover, NADES can be modified to improve the extraction yield of target analytes at relatively low prices [20]; thus, NADES are employed to extract bioactive components from plants.

Currently, researchers are investigating a greener, safer, and cleaner method using the combination of UAE and NADES that significantly improves the extraction efficiency of bioactive compounds, such as crocins from gardenia fruits [21], phenolics from peels of *Carya cathayensis* Sarg [20], anthocyanins from wine lees [22], and bioactive compounds from apple pomace [23]. Lei Jiang et al. used ethanol to extract phenolic compounds from bamboo shoot shells, and this research indicated that 58% ethanol was the optimal concentration to reach the highest extraction yield and antioxidant activity [24]. Pengfei Zhou et al. used NADES-based UAE to enhance the phenolic extraction from the *Morus alba L.* leaves. NADES prepared from citric acid/choline chloride have the highest extraction yield, and the optimal UAE conditions were also discovered [25]*.* Chun Chen et al. found optimal conditions for the UAE of polysaccharides from black mulberry fruits (*Morus nigra L.)* and demonstrated the biological activities of these components.

However, the extraction yield of biological compounds from plants relies on numerous factors, such as the physiochemical attributes of plant materials, extraction conditions, and the nature of solutes [24]. Previous studies concentrated on evaluating the extraction yield of phenolics and polysaccharides from mulberry leaves and fruits, respectively. It is preferable to discover optimal extraction conditions to reach the maximal extraction efficiency of phenolics and flavonoids from black mulberry fruits owing to the differences in plant material and the nature of biological compounds. Moreover, the previous study did not simultaneously show the extraction efficiency of these compounds and the variation of antioxidant activities during NADES-based UAE. The simultaneous optimization of antioxidant activities, flavonoid, and phenolic recovery was not examined*.*

Therefore, this research evaluated the extractability of different NADES combined with UAE to extract bioactive compounds (phenolics and flavonoids) from the black mulberry fruit. The effect of NADES-based UAE factors (liquid-to-solid ratio (LSR), water content in NADES, temperature, and time) on antioxidant activities, total phenolic and flavonoid contents (TPC and TFC, respectively) were determined by one-factor experiments. Response surface methodology (RSM) with the Box-Behnken design (BBD) model was used to optimize the NADES-based UAE process. The morphological variation of the black mulberry fruit powder surface with and without sonication was examined using scanning electron microscopy (SEM).

## Materials and methods

### Materials

Black mulberry fruits were purchased from DALAT FARM company, Da Lat, Lam Dong, Vietnam, and washed to remove soil. Black mulberry fruits were dried at 45 °C for 40 h and milled to attain black mulberry fruit powder (BMP), having 8% moisture content. Hydrochloric acid (purity ≥ 36.5%), sodium carbonate (purity ≥ 99.5%), iron (II) sulfate heptahydrate (purity ≥ 99%), absolute ethanol (purity ≥ 99.8%), salicylic acid (purity ≥ 99%), hydrogen peroxide (purity 34.5–36.5%) (aluminum chloride hexahydrate (purity 99%), 1,1-diphenyl-2-picrylhydrazyl (DPPH, purity ≥ 97%), Folin–Ciocalteu reagent (concentration 1.9–2.1N), gallic acid monohydrate (purity ≥ 98%), sodium acetate trihydrate (purity ≥ 99%), potassium chloride (purity ≥ 99%), potassium acetate (purity ≥ 99%), 2-Azino-bis (3-ethylbenzothiazoline-6-sulfonic acid) diammonium salt (ABTS, purity ≥ 98%), rutin hydrate (purity ≥ 94%), and 6-hydroxy-2,5,7,8-tetramethylchroman-2-carboxylic acid (Trolox, purity 98%) were purchased from Sigma-Aldrich Chemical Co., Ltd, Singapore, Singapore. The chemicals used to prepare NADES were purchased from HiMedia Laboratories, Mumbai, Maharashtra, India.

### Preparing and screening NADES

NADES were produced using a heating method [26]. The hydrogen bonding donors and acceptors were blended in the appropriate molar ratio (Table 1), the mixtures were heated at 90 °C and stirred using a magnetic machine (model: C—MAG HS 7, IKA Industrie, Humboldtstraße, Königswinter, Germany). NADES are produced when a homogeneous and transparent liquid is formed. The molar ratios, chemicals, and acronyms of the NADES are listed in Table 1.

**Table 1:** NADES prepared in this research

No	Abbreviation	HBD	HBA	Molar ratio
1	NADES1	Citric acid	1,2–Propanediol	2:1
2	NADES2	Citric acid	Choline chloride	2:1
3	NADES3	Citric acid	Glycerin	2:1
4	NADES4	Citric acid	Glucose	2:1
5	NADES5	Lactic acid	1,2–Propanediol	2:1
6	NADES6	Lactic acid	Glycerin	2:1
7	NADES7	Lactic acid	Glucose	2:1
8	NADES8	Lactic acid	Choline chloride	2:1
9	NADES9	Acetic acid	Glycerin	2:1
10	NADES10	Acetic acid	Glucose	2:1
11	NADES11	Acetic acid	1,2–Propanediol	2:1
12	NADES12	Tartaric acid	Choline chloride	2:1
13	NADES13	Tartaric acid	Glycerin	2:1
14	NADES14	Tartaric acid	1,2–Propanediol	2:1
15	ACE60	Aqueous acetone solution (60%)		

BMP was weighed at 0.8333 ± 0.0010 g in an amber glass jar, and 25 ml of NADES (20% water content, g/g) or ACE60 was added to BMP. Then, the mixture was sonicated in an ultrasonic bath (model: S300H, Elma Schmidbauer, Gottlieb-Daimler-Straße, Hohentwiel, Germany, ultrasonic power: 300W) at 70 °C for 15 min of extraction time. The mixtures were centrifuged at 1800 g at 30 °C for 20 min (DM0412, DLAB Scientific Co., Ltd, Shunyi, Beijing, China) to separate the solid. After that, the TPC and TFC of the extracts were quantified, and ACE60 was used as the control solvent.

### One-factor experiments for UAE combined with NADES

The appropriate NADES were chosen from Sect. "Preparing and screening NADES" for use in one-factor experiments. One-factor experiments were performed to investigate the influence of the technical parameters, including liquid-to-solid ratios (LSR (NADES: BMP), 10–100 ml/g), water content (10, 20, 30, 40, and 50%, g/g), temperature (30, 40, 50, 60, 70, and 80 °C), and extraction time (5, 10, 15, 30, 50, 70, and 90 min) on the efficiency of UAE process. Briefly, the quantified amount of BMP was added to a 100-ml amber glass bottle, 25 ml of NADES were poured into an amber glass bottle, and then sonicated under selected conditional ranges. The mixtures were centrifuged at 1800 g at 30 °C for 20 min to eliminate solids and quantify TPC, TFC, and antioxidant activities.

### Optimization of NADES-based UAE process

The BBD model was employed to perform the optimization of the UAE process. The surveyed ranges of variables (LSR, water content in NADES, temperature, and extraction time) for optimization were employed from one-factor experiments and three levels: high (+ 1), mid (0), and low (− 1), corresponding to the upper, appropriate, and lower conditions, respectively. The highest extraction yield for each response in the one-factor experiments was obtained under the appropriate conditions, whereas the lower and upper conditions were the boundary values. Twenty-nine experiments with five center points were performed, and the results were used to calculate the statistical parameters. Linear, interactive, and quadratic terms were determined and expressed in polynomial regression models, Eq. (1):

1 $$Y={b}_{0}+\sum_{a=1}^{m}{b}_{i}{X}_{a}+\sum_{a=1}^{m}{b}_{ii}{X}_{a}^{2}+\sum_{a=1}^{m}\sum_{b=1}^{m}{b}_{ij}{X}_{a}{X}_{b}$$

in which: b_o_, b_i_, b_j_, and b_ij_ are the regression coefficients of the intercept point, linear, squared, and interaction effects; k is the number of independent variables (k = 4). Y represents the responses, and X symbolizes the values of the variables. The detailed experimental design of four independent variables is presented in Table 2. The responses (Y) were TPC (mg GAE/g db), TFC (mg RE/ g db), ABTS (mM TE/g db), OH (mM TE/g db), and DPPH (µM TE/g db).

**Table 2:** Experimental design

Independent factors	Units	The value of independent factors		
		Low (− 1)	Middle (0)	High (+ 1)
X_1_: LSR	mL/g	50	60	70
X_2_: Water content	%	30	40	50
X_3_: Temperature	°C	60	70	80
X_4_: Time	min	10	15	30

ANOVA was performed to quantify the significant statistical differences in the regression model for each response. The fit of the forecast models was validated using F-values and p-values (p < 0.05). The coefficients of adjusted determination and determination (adjusted R^2^ and R^2^, respectively) were used to demonstrate the quality of forecast models. The prediction error (%) was employed to compare the distinction between the forecast and experimental results and was calculated using Eq. (2).

2 $$Prediction\,error=\frac{\left|the\,mean\,of\,measured\,value-predicted\,values\right|}{the\,mean\,of\,measured\,value}*100$$

### The quantification of antioxidant activities, total phenolic, and total flavonoid contents

TPC was quantified using Folin-Ciocalteu reagent, and TFC was measured using aluminum hydroxyl as the reagent [26]. TPC (y = 0.0031x-0.0026; R^2^ = 0.9940; y: absorbance; x: gallic acid contents (mg/L)) and TFC (y = 0.0022x-0.0014; R^2^ = 0.9995; y: absorbance; x: rutin contents (mg/L)) were shown as milligram gallic acid equivalent per gram of dry basis (mg GAE/g db) and milligram rutin equivalent per gram of dry basis (mg RE/g db), respectively. The antioxidant activities of the extracts were quantified using colorimetric methods, and Trolox was used to construct the standard curves. DPPH radical quenching capacity (DPPH) was determined by the LarsMüller method [16] using an ethanolic DPPH solution, and ABTS^+^ radical scavenging capacity (ABTS) was determined by the Lingfeng Wu method using an aqueous ABTS^+^ working solution [26]. The hydroxyl radical quenching capacity (OH) was quantified by the Hongjie Yuan method [27] using salicylic acid as the indicator. The DPPH radical quenching capacity was expressed as micromole Trolox equivalent per gram of dry basis (µM TE/g db), while hydroxyl radical and ABTS^+^ quenching capacities were shown as millimole Trolox equivalent per gram of dry basis (mM TE/g db).

### Surface morphology of black mulberry fruit powder

The surface morphology of BMP with and without sonication was determined using scanning electron microscopy (SEM, model: Prisma E SEM, ThermoFisher Scientific, Waltham, Massachusetts, America). The sample preparation procedure was based on the Sujata S. Patil method [28]. Samples were put on carbon tape to preclude the loss of samples and enclosed on the sample plate. Then, the samples were covered with a thin layer of gold metal. The procedure was conducted under vacuum; then, the samples were relocated and directly monitored under SEM at distinctive magnifications and 5 kV.

### Statistical analysis

All experiments were performed threefold and shown as the mean ± SD. Statistical analysis was conducted by Minitab 19 (Minitab, Inc., Pennsylvania, USA). Fisher tests and analysis of variance (ANOVA) were performed to compare statistically significant differences among the experimental results with 95% confidence. The BBD model was performed using Design-Expert v.13 software (Stat-Ease Inc., Minneapolis, Minnesota 55413, USA), and the linear, quadratic, and interaction regression coefficients were fitted to second-order polynomial regression models. Graphics were built by Origin Pro (Origin Lab, Northampton, Massachusetts, USA).

## Results and discussions

### Evaluating the extraction performance of NADES

**Fig. 1:**
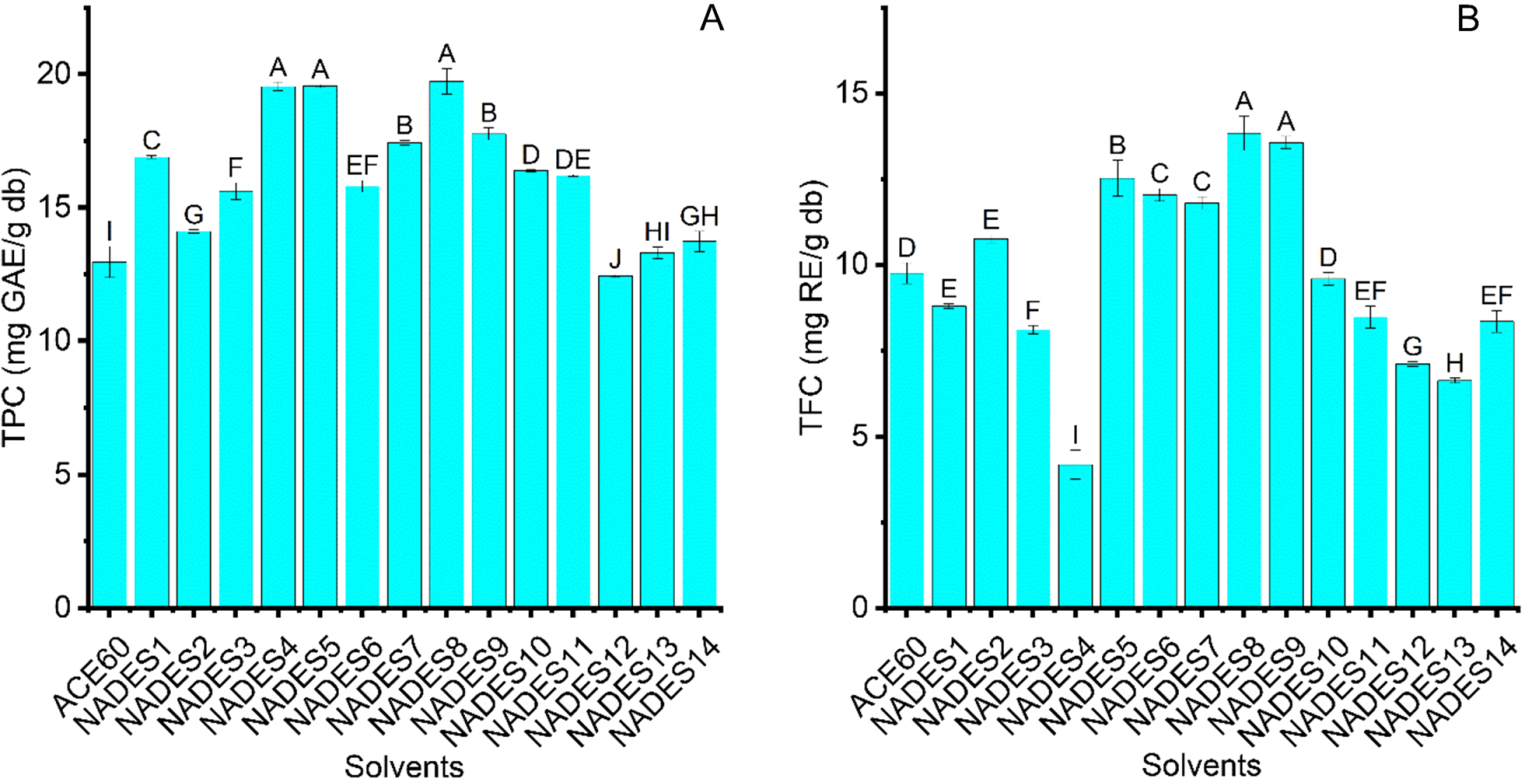
The extraction performance of fourteen distinctive solvents (NADES) and acetone solution (60%, v/v) at LRS: 30 ml/g, 20% water content, and temperature: 70°C for 15 min; **A** TPC; **B** TFC. Distinctive letters (**A**, **B**, **C**, **D**, **E**, **F**, **G**, **H**, **I**) showed significant statistical differences

NADES can dissolve plant cell walls by forming intermolecular interactions with cellulose chains [28]. Extraction yield directly correlates with NADES viscosity and polarity, relying on their components, water content, and the molar ratio of HBA and HBD [13, 28]. The extractability of 14 selected NADES (Table 1) and acetone solution (60%) under sonication was evaluated through TPC and TFC, and the results are expressed in Fig. 1A-B. There were considerable differences in the TPC and TFC of BMP extracts attained by 14 NADES and ACE60 combined with UAE (p < 0.05). When NADES4, NADES5, and NADES8-based UAE were employed, the extraction yield of phenolics was the highest at 19.54 ± 0.16, 19.56 ± 0.06, and 19.74 ± 0.48 mg GAE/g db, respectively, followed by NADES9. Under sonication, TFC attained using NADES as a solvent was in the range of 4.18 ± 0.53 to 13.84 ± 0.50 mg RE/g db, while that of ACE60 was 9.75 ± 0.30 mg RE/g db. The extraction yield of flavonoids was the highest when using NADES5, NADES8, and NADES9-based UAE. The dissolution capacity of target analytes in solvents can comply with the “like dissolve like” rule that decides the extraction yield of phenolics and flavonoids [29, 30]. NADES4, NADES5, NADES8, and NADES9 had a higher extractability of phenolics and flavonoids than ACE60, possibly due to the polar approximation between these NADES and bioactive compounds in BMP [29]. Additionally, the higher extraction capacity of these NADES can stem from the intermolecular hydrogen interaction of NADES with phenolics and flavonoids in BMP [31]. However, NADES4 combined with UAE showed the lowest extraction yield of flavonoids in BMP compared to ACE60, possibly due to their high viscosity. The high viscosity of NADES4, resulting from an extensive hydrogen-bonding network between HBD and HBA, can hamper the diffusivity and mass transfer [31]. In contrast, the higher phenolic extraction performance of NADES4 than flavonoids can be owing to the strong hydrogen-bonding interaction between NADES4 and phenolic compounds. This interaction can improve the extraction yield of phenolics even with the high viscosity of NADES4 [32, 33]. Therefore, NADES8 was suitable for extracting phenolics and flavonoids from BMP.

### One-factor experiments for UAE combined with NADES8

#### ***E***ffect of*** liquid-to-solid ratios***

**Fig. 2:**
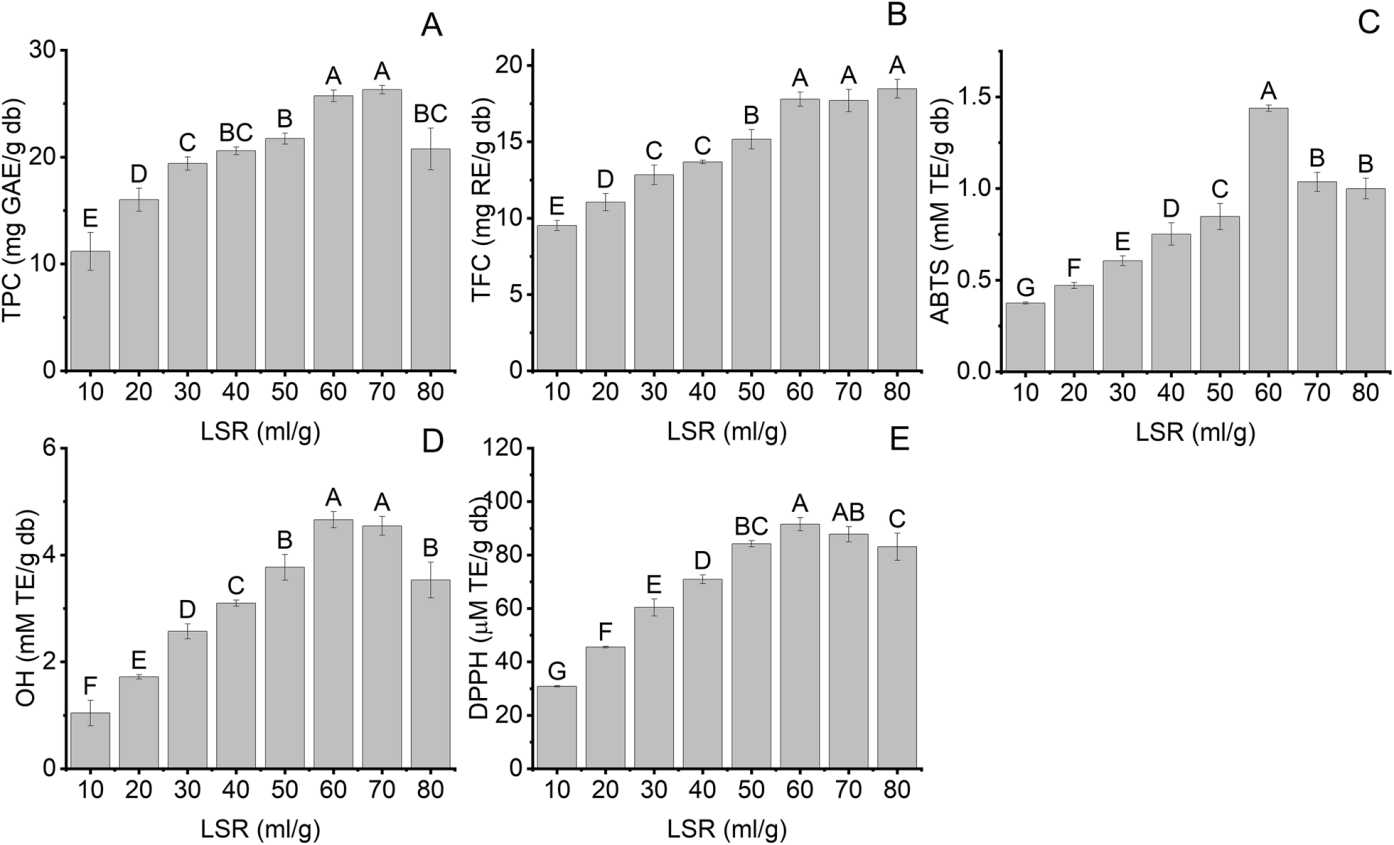
The effect of LSR on the extraction yield of bioactive compounds and their antioxidant activity at water content (20%), 70°C, and extraction time (15 min); **A** TPC; **B** TFC; **C** ABTS; **D** OH; **E** DPPH. Distinctive letters (**A**, **B**, **C**, **D**, **E**, **F**, **G**) showed significant statistical differences

In light of an economic perspective, LSR is the essential parameter influencing the running cost of extraction factories [23]. Therefore, the effect of LSR (10–80 ml/g) on the extraction yield of bioactive compounds and antioxidant activity using UAE combined with NADES8 was examined, and the results are illustrated in Fig. 2A–E. TPC and TFC increased by 2.3 and 1.9 times when LSR rose from 10 to 60 ml/g (Fig. 2A, B). It can be explained that increasing LSR enhances the mass transfer capacity due to reducing the viscosity of the extraction medium [13]. The high LSR can generate a significant difference in concentrations between BMP and medium, which probably facilitates the release of phenolics and flavonoids from the cell matrix of BMP into the NADES8 medium. Moreover, when LSR increases, the vicious reduction can drop the cavitation threshold, which can help the cavitation phenomenon quickly occur [34]. The cavitation threshold defines a minimum acoustic pressure needed to develop a cavity in the liquid environment during the expansion cycle; this phenomenon can strengthen the cavitation effect within an extraction medium [28]. The high LSR increases the contact area between BMP and the extractant as well as can be combined with ultrasonic irradiation, which can contribute to more fragmentation of BMP, sonoporation, and erosion on the BMP surface [35]. The combination of these phenomena can improve the extraction yield of bioactive compounds from BMP. When the SLR was lower than 60 ml/g, the high viscosity of the extraction medium can hinder the cavitation effects, resulting in lowering extraction yield [35]. However, when LSR was more extensive than 60 ml/g, there was a decline in TPC, whereas TFC remained unchanged. The decrease in the TPC can be ascribed to the rising cavitation effect leading to the deterioration of phenolics [35]. Rubiya Rashid et al. (2023) also investigated the effect of LSR on the recovery yield of phenolics and flavonoids from apple pomaces. In this study, when LSR escalated to 30 ml/g, phenolics, and flavonoid contents rose to 5.8 mg GAE/g. Nevertheless, with a further rise in LSR to 50 ml/g, a downward trend was witnessed in TPC acquired from apple pomace [23].

The ABTS^+^, DPPH, and hydroxyl radical quenching capacities were performed, and the results are expressed in Fig. 2C–E. When the LSR increased from 10 to 60 ml/g, ABTS, OH, and DPPH witnessed an increase of 3.86, 4.42, and 2.96 times, respectively. However, further increase in LSR to 80 ml/g, ABTS, OH, and DPPH were declined by 1.4, 1.3, and 1.1 times, respectively, compared to 60 ml/g of LSR. It can be explained that the radical quenching capacities have a positive relationship with TPC and TFC [26]. The positive correlation between TPC, TFC, and antioxidant activities was investigated by Oscar Zannou et al., who employed the NADES-based UAE to recover anthocyanin from blackberry [31]. Therefore, to ensure the highest extraction yield of phenolics and flavonoids and to save solvent volumes, 60 ml/g was the suitable LSR for UAE combined with NADES8 to attain the highest extraction yield of bioactive compounds from BMP.

#### Effect of water content in NADES8

**Fig. 3:**
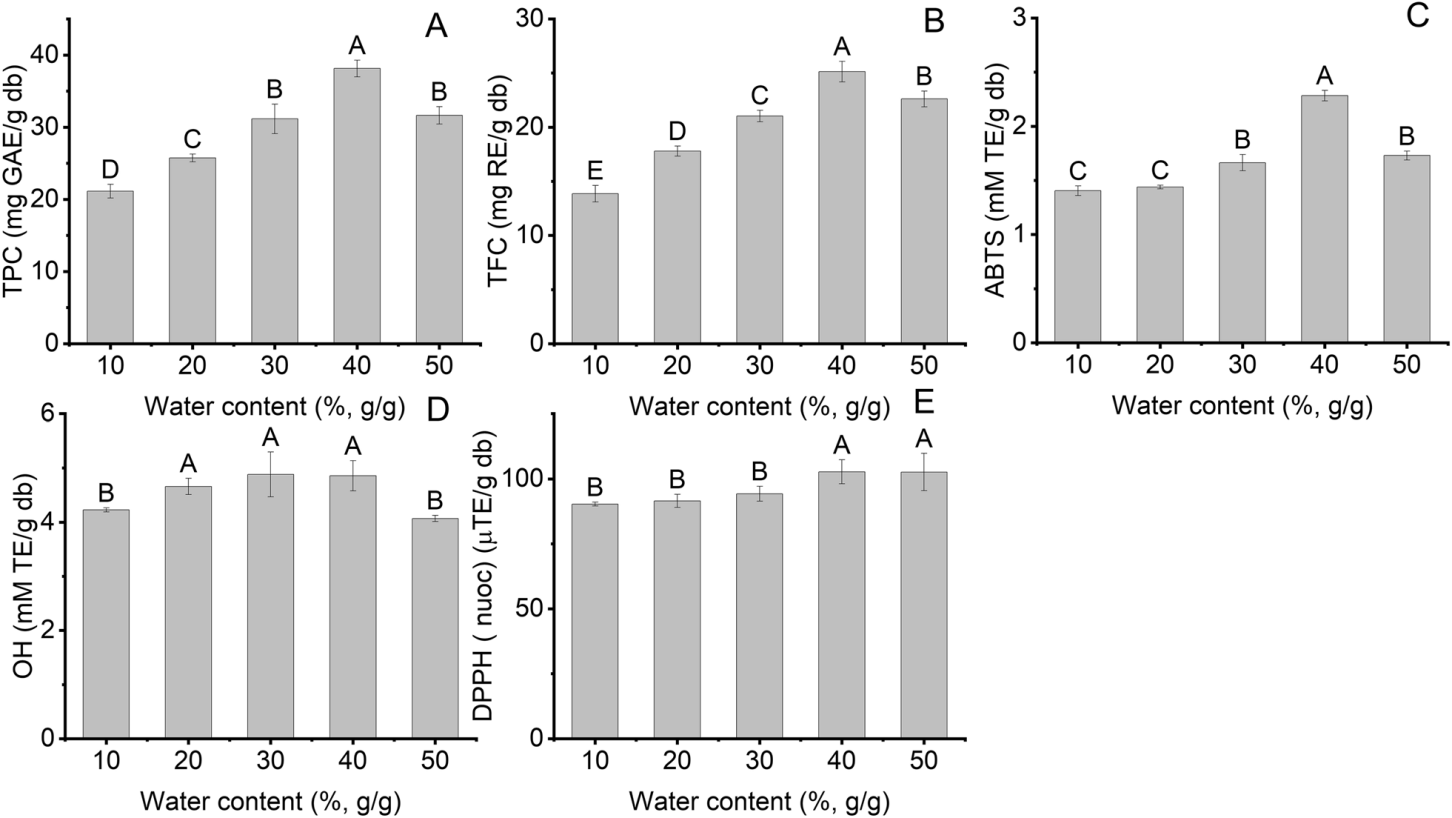
The effect of water content in NADES on the extraction yield of bioactive compounds and their antioxidant activity at LSR (60 ml/g), 70°C and extraction time (15 min); **A** TPC; **B** TFC; **C** ABTS; **D** OH; **E** DPPH. Distinctive letters (**A**, **B**, **C**, **D**, **E**) showed significant statistical differences

Water content in NADES8 plays an integral part in extraction efficiency owing to influencing the viscosity and polarity of the NADES [28]. The effect of water content on TPC, TFC, and antioxidant activity was surveyed from 10 to 50% under sonication, and the results are demonstrated in Fig. 3A, E. As can be seen in Fig. 3A, B, there was a significant increase in TPC and TFC by 1.8 times at 40% compared to 10% water content. Increased water addition can reduce the NADES8 viscosity, promoting the cavitation effect. The enhancement of the cavitation effect can generate greater shear force and turbulence that facilitate mass transfer. This phenomenon can accelerate the release of phenolics and flavonoids in BMP into NADES8, leading to an increase in extraction yield [28, 36]. However, with a further increase in water content to 50%, the UAE of phenolics and flavonoids decreased by 1.2 and 1.1 times, respectively. It can be explained that excessively adding water destroys hydrogen-bonding networks between NADES8 and bioactive compounds in BMP, leading to a decrease in TPC and TFC [28]. This result reached an agreement with Zeng et al. who employed UAE combined with NADES to acquire bioactive compounds from Chinese wild rice [37].

The ABTS, DPPH, and ABTS were investigated, and their values are shown in Fig. 3C–E. When the water content grew from 10 to 40%, ABTS, OH, and DPPH experienced an increase of 1.6, 1.2, and 1.1 times, respectively. However, with a further rise in water content to 50%, ABTS and OH dropped by 1.3 and 1.2 times, respectively, whereas DPPH stabilized compared to 40% water content. Therefore, the water content in NADES8 was 40% suitable for NADES8-based UAE to acquire the highest extraction yield of bioactive compounds from BMP.

#### ***E***ffect of temperature

**Fig. 4:**
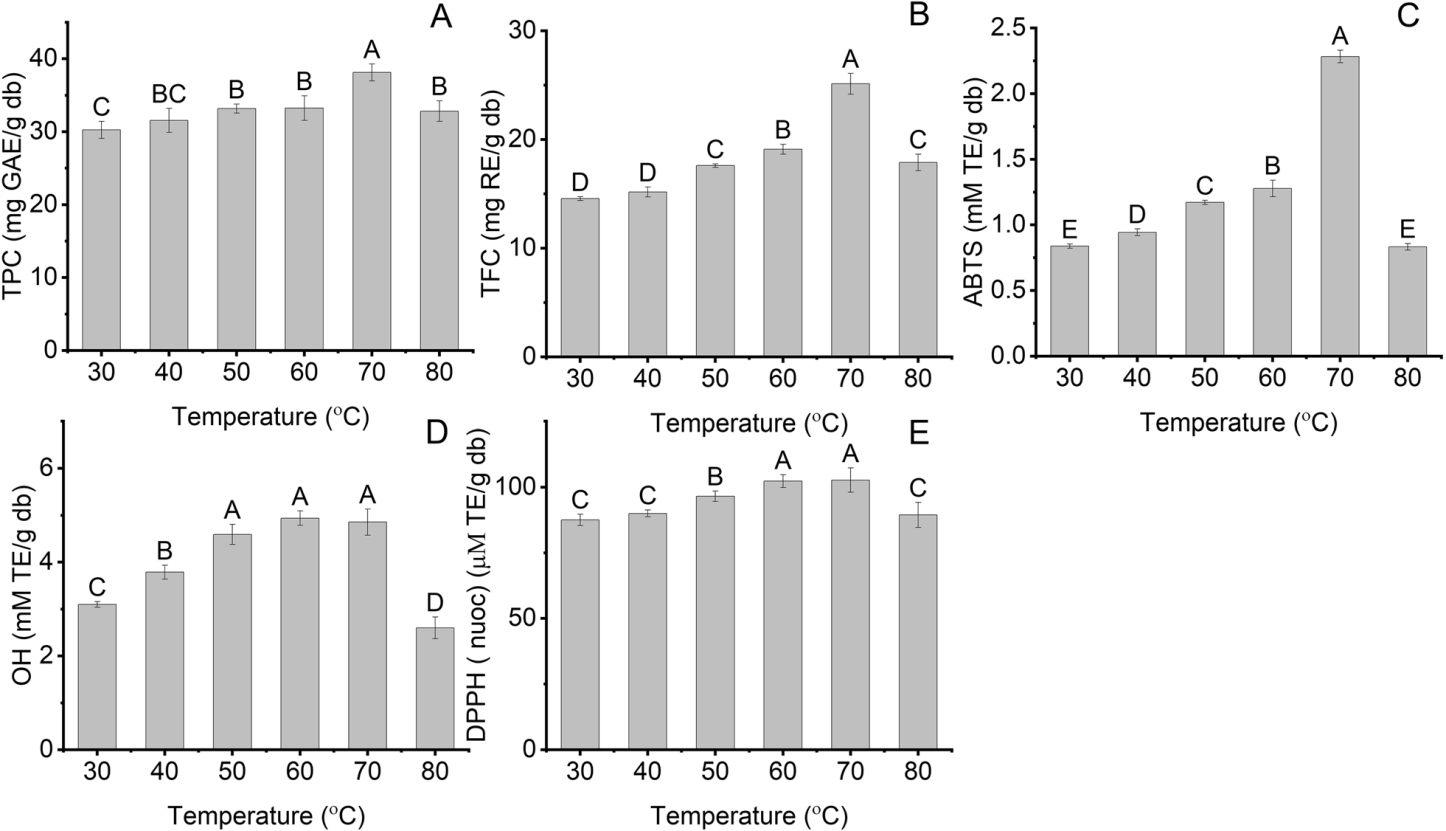
The effect of temperature on the extraction yield of bioactive compounds and their antioxidant activity at LSR (60 ml/g), 40% water content, and extraction time (15 min); **A** TPC; **B** TFC; **C** ABTS; **D** OH; **E** DPPH. Distinctive letters (**A**, **B**, **C**, **D**, **E**) showed significant statistical differences

Temperature is one of the main elements affecting the extraction yield of bioactive compounds in BMP because temperature changes the viscosity of NADES8 [23]. Thus, the effect of temperature on the extraction yield of bioactive compounds and antioxidant activity using NADES8 combined with UAE was evaluated in the range of 30 to 80°C. As shown in Fig. 4A, B, TPC and TFC were higher 1.2 and 1.7 times, respectively, at 70 °C than 30 °C. The escalation in temperature can strengthen the desorption capacity and solubility of analytes, resulting in the enhancement of solvent diffusivity into the cell matrix [13]. Moreover, an increase in temperature can decrease the viscosity of NADES8, possibly improving the mass transfer process that can enhance the extraction yield of bioactive compounds in BMP [38]. On the other hand, a further increase in temperature to 80 °C decreased TPC and TFC to 32.82 ± 1.41 mg GAE/g db and 17.9 ± 0.76 mg RE/g db, respectively. The phenomenon can be attributed to a drop in surface tension that impairs the intensity of the cavitation effect. Decreasing the intensity of the cavitation effect can be the main reason for reducing the intensity of collapsing bubbles that impair mass transfer, lowering the release of phenolics and flavonoids in BMP into NADES8. A high temperature over 70 °C can also deteriorate phenolics and flavonoids; the synergistic effect of the two phenomena can decrease the extraction yield of bioactive compounds from BMP [13, 39]. Soumen Dey and Virendra K. Rathod et al. (2013) explored a similar trend by employing UAE to obtain β-carotene from *spirulina platensis* [40]. Figure 4C–E shows the antioxidant activities of BMP extracts obtained using NADES8-based UAE. ABTS, OH, and DPPH were increased by 2.7, 1.6, and 1.2 times at 70 °C in comparison with their values at 30 °C. However, the reverse trend was confirmed when the temperature increased to 80 °C. Therefore, the extraction temperature of 70 °C was the proper condition for NADES8-based to get the highest extraction yield of bioactive compounds from BMP.

#### Effect of time

**Fig. 5:**
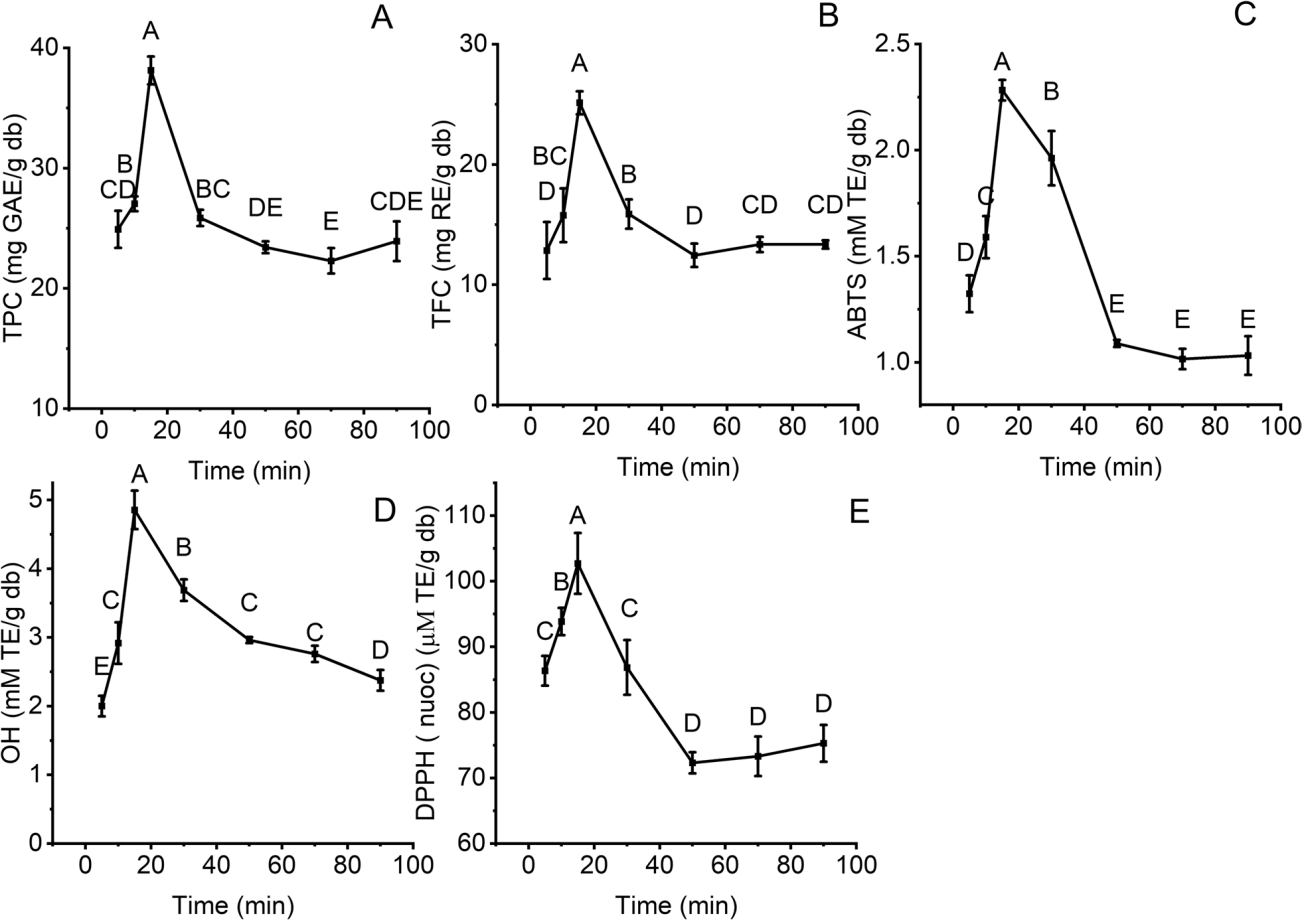
The effect of time on the extraction yield of bioactive compounds and their antioxidant activity at LSR (60 ml/g), 40% water content, and 70°C; **A** TPC; **B** TFC; **C** ABTS; **D** OH; €: DPPH. Distinctive letters (**A**, **B**, **C**, **D**, **E**) showed significant statistical differences

Extraction time can impact the extraction yield of bioactive compounds and antioxidant activities from BMP [35]; therefore, the influence of extraction time on TPC, TFC, and antioxidant activity using NADES8-based UAE was investigated at 5, 10, 15, 30, 50, 70 and 90 min (Fig. 5A–E). TPC and TFC initially increased by 1.5 and 2.0 times as the extraction time rose from 5 to 15 min (Fig. 5A, B). The high extraction rate can be attributed to a high concentration distinction between BMP and NADES8, which can shorten extraction time [41]. Additionally, the shockwaves released from the collapsing cavitation bubbles erode the cell walls of BMP. This effect can promote the solubility and diffusivity of bioactive compounds in the extractant, which improves extraction yield [13]. However, when extraction time was continuously prolonged, the extraction yield of bioactive compounds declined. This observation highlights that extended exposure of BMP to ultrasound can trigger the degradation of phenolics and flavonoids [13]. The deterioration of these compounds results in the reduction in ABTS, DPPH, and OH of BMP extracts [42]. This trend was similar to our previous study, which employed NADES-based UAE to extract phenolics and flavonoids from mangosteen rinds [43]. As a result, fifteen minutes was an appropriate extraction time to obtain the highest extraction yield of bioactive compounds from BMP using NADES8-based UAE.

### Optimizing ultrasonic-assisted extraction of bioactive compounds in BMP

#### The regression models

Twenty-nine experiments were conducted to examine the influence of four independent variables (LSR, water content in NADES8, temperature, and extraction time) on the responses (TPC, TFC, ABTS, OH, and DPPH). The optimization results of responses are expressed in Table 3, and Table 4 shows regression analysis results. Second-order polynomial regression models were employed to express suggested models after analyzing the multiple regression of the experimental results. The second-order polynomial equations showed the correlation between independent variables and responses, expressed in Eqs. (3) to (7):

**Table 3:** The optimization results of the NADES-based UAE process

		Factor					TPC		TFC		ABTS		OH		DPPH	
	Run	X_1_	X_2_		X_3_	X_4_	Forecast values	Experimental values	Forecast values	Experimental values	Forecast values	Experimental values	Forecast values	Experimental values	Forecast values	Experimental values
	1	0	− 1		− 1	0	24.4	24.9 ± 0.4	14.7	14.6 ± 0.4	0.87	0.83 ± 0.10	7.4	7.2 ± 0.4	161	164 ± 7
	2	0	0		− 1	1	32.5	32.7 ± 1.0	14.3	16.0 ± 1.0	1.02	1.00 ± 0.10	9.8	10.1 ± 0.2	175	176 ± 13
	3	0	1		− 1	0	29.9	30.7 ± 1.5	17.2	16.9 ± 1.5	1.03	0.99 ± 0.09	8.3	8.6 ± 1.2	171	161 ± 9
	4	0	0		− 1	− 1	24.2	25.8 ± 2.0	13.4	13.7 ± 2.0	0.76	0.97 ± 0.10	5.5	5.8 ± 0.7	165	167 ± 18
	5	− 1	0		− 1	0	31.8	29.6 ± 1.1	15.2	15.3 ± 1.1	0.80	0.81 ± 0.08	7.8	7.6 ± 0.2	140	149 ± 8
	6	1	0		− 1	0	31.4	30.7 ± 2.3	16.6	15.1 ± 2.3	1.07	0.99 ± 0.11	7.2	6.8 ± 2.3	194	189 ± 4
	7	0	1		0	1	24.7	24.1 ± 0.9	13.4	12.2 ± 1.2	1.99	1.97 ± 0.05	10.1	9.9 ± 0.2	180	183 ± 11
	8	1	0		0	− 1	23.3	23.2 ± 3.1	14.4	13.9 ± 0.4	1.50	1.64 ± 0.18	7.1	7.7 ± 0.2	194	195 ± 8
	9	0	− 1		0	− 1	13.7	12.3 ± 0.1	10.8	11.6 ± 2.4	1.49	1.48 ± 0.25	7.0	7.0 ± 0.4	163	167 ± 6
	10	1	1		0	0	29.5	28.9 ± 0.4	16.4	17.9 ± 1.4	1.78	1.75 ± 0.17	8.9	8.4 ± 0.2	186	198 ± 13
	11	0	0		0	0	37.8	37.8 ± 0.7	25.5	25.5 ± 1.3	2.28	2.28 ± 0.05	4.9	4.9 ± 0.3	99	99 ± 6
	12	− 1	0		0	− 1	24.2	24.6 ± 1.0	7.6	6.8 ± 0.6	1.74	1.53 ± 0.02	6.9	6.8 ± 0.2	145	145 ± 4
	13	1	− 1		0	0	26.2	26.4 ± 0.6	13.8	14.8 ± 1.6	2.00	1.80 ± 0.16	8.8	8.6 ± 0.3	207	202 ± 18
	14	0	0		0	0	37.8	37.8 ± 0.7	25.5	25.5 ± 1.3	2.28	2.28 ± 0.05	4.9	4.9 ± 0.3	99	99 ± 6
	15	0	− 1		0	1	31.3	30.3 ± 4.0	11.0	10.2 ± 0.6	1.82	1.97 ± 0.08	10.0	10.0 ± 0.2	174	181 ± 16
	16	0	0		0	0	37.8	37.8 ± 0.7	25.5	25.5 ± 1.3	2.28	2.28 ± 0.05	4.9	4.9 ± 0.3	99	99 ± 6
	17	1	0		0	1	34.8	35.7 ± 1.1	11.7	11.5 ± 0.9	2.15	2.36 ± 0.44	10.3	10.8 ± 0.6	208	207 ± 9
	18	− 1	1		0	0	26.7	27.1 ± 3.8	12.4	12.8 ± 0.6	2.09	2.31 ± 0.12	8.6	8.6 ± 0.1	161	160 ± 17
	19	0	0		0	0	37.8	37.8 ± 0.7	25.5	25.5 ± 1.3	2.28	2.28 ± 0.05	4.9	4.9 ± 0.3	99	99 ± 6
	20	0	0		0	0	37.8	37.8 ± 0.7	25.5	25.5 ± 1.3	2.28	2.28 ± 0.05	4.9	4.9 ± 0.3	99	99 ± 6
	21	− 1	− 1		0	0	25.2	26.6 ± 2.9	12.1	12.0 ± 1.3	1.41	1.47 ± 0.04	7.8	8.1 ± 1.0	123	104 ± 13
	22	− 1	0		0	1	30.0	31.3 ± 2.7	12.7	12.1 ± 0.5	1.64	1.51 ± 0.24	9.1	8.8 ± 0.1	149	148 ± 6
	23	0	1		0	− 1	24.9	24.0 ± 1.4	11.3	11.7 ± 3.2	1.79	1.61 ± 0.11	7.8	7.5 ± 0.1	174	174 ± 12
	24	− 1	0		1	0	24.7	23.5 ± 0.6	10.4	11.6 ± 0.1	1.03	1.09 ± 0.03	1.8	2.0 ± 0.6	100	111 ± 8
	25	0	0		1	1	28.1	27.2 ± 0.6	11.2	12.2 ± 0.7	1.12	0.93 ± 0.02	3.5	3.1 ± 0.4	135	126 ± 4
	26	0	1		1	0	22.0	22.8 ± 0.4	12.8	11.9 ± 0.3	1.19	1.26 ± 0.15	3.2	3.8 ± 0.3	130	126 ± 6
	27	1	0		1	0	28.9	29.2 ± 0.6	14.8	14.5 ± 0.2	1.03	1.00 ± 0.02	3.8	3.8 ± 0.4	154	151 ± 20
	28	0	0		1	− 1	19.0	19.5 ± 0.6	9.8	9.5 ± 0.3	0.84	0.88 ± 0.02	2.5	2.1 ± 0.5	127	120 ± 4
	29	0	− 1		1	0	22.7	23.1 ± 1.2	12.4	11.6 ± 0.3	0.90	0.97 ± 0.11	3.2	3.3 ± 0.7	123	134 ± 22

**Table 4:** The ANOVA results of the optimization process

	Coefficient	TPC	TFC	ABTS	OH	DPPH
Intercept	b_0_	37.8**	25.5**	2.280**	4.9**	99.4**
Linear	b_1_	0.9*	1.4**	0.068	0.3*	27.1**
	b_2_	1.2**	0.7*	0.115*	0.2	4.2
	b_3_	− 2.4**	− 1.7**	0.047	− 2.3**	− 19.8**
	b_4_	4.3**	0.6	0.135*	1.3**	4.5
Interaction	b_12_	0.5	0.6	− 0.225*	− 0.2	− 14.7**
	b_13_	1.1	0.8	− 0.070	0.6**	0.1
	b_14_	1.4*	− 1.9**	0.188*	0.3	2.4
	b_23_	− 1.6*	− 0.5	0.033	− 0.2	− 1.0
	b_24_	− 4.5**	0.5	− 0.033	− 0.2	− 1.2
	b_34_	0.2	0.1	0.004	− 0.8**	− 0.6
Quadratic	b_11_	− 3.2**	− 5.9**	− 0.237**	1.6**	35.3**
	b_22_	− 7.7**	− 5.9**	− 0.221**	2.0**	34.6**
	b_33_	− 5.4**	− 5.3**	− 1.060**	− 1.4**	12.2**
	b_44_	− 6.5**	− 8.0**	− 0.287**	1.8**	39.0**
Degree of freedom		14	14	14	14	14
F-values		49.48	45.71	21.5	71.13	27.31
p-values		< 0.0001	< 0.0001	< 0.0001	< 0.0001	< 0.0001
R^2^		0.9802	0.9786	0.9556	0.9861	0.9647
Adjusted R^2^		0.9604	0.9572	0.9111	0.9723	0.9293

*significant differences (p < 0.05)

**highly significant differences (p < 0.01)

3 $${Y}_{TPC}=37.8+0.9{X}_{1}+1.2{X}_{2}-2.4{X}_{3}+4.3{X}_{4}+1.4{X}_{1}{X}_{4}-1.6{X}_{2}{X}_{3}-4.5{X}_{2}{X}_{4}-3.2{X}_{1}^{2}-7.7{X}_{2}^{2}-5.4{X}_{3}^{2}-6.5{X}_{4}^{2}$$

4 $${Y}_{TFC}=25.5+1.4{X}_{1}+0.7{X}_{2}-1.7{X}_{3}-1.9{X}_{1}{X}_{4}-5.9{X}_{1}^{2}-5.9{X}_{2}^{2}-5.3{X}_{3}^{2}-8.0{X}_{4}^{2}$$

5 $${Y}_{ABTS}=2.28+0.12{X}_{2}-0.14{X}_{4}-0.23{X}_{1}{X}_{2}+0.19{X}_{1}{X}_{4}-0.24{X}_{1}^{2}-0.22{X}_{2}^{2}-1.06{X}_{3}^{2}-0.29{X}_{4}^{2}$$

6 $${Y}_{OH}=4.9+0.3{X}_{1}-2.3{X}_{3}+1.3{X}_{4}+0.6{X}_{1}{X}_{3}-0.8{X}_{3}{X}_{4}+1.6{X}_{1}^{2}+2.0{X}_{2}^{2}-1.4{X}_{3}^{2}+1.8{X}_{4}^{2}$$

7 $${Y}_{DPPH}=\,99.4+27.1{X}_{1}-19.8{X}_{3}-14.7{X}_{1}{X}_{2}+35.3{X}_{1}^{2}+34.6{X}_{2}^{2}+12.2{X}_{3}^{2}+39.0{X}_{4}^{2}$$

The results of ANOVA can demonstrate the confidence of second-order polynomial regression models (Table 3). Such models were highly significant (p < 0.0001) for determining TPC, TFC, ABTS, OH, and DPPH. The predicting models were considerably proper for F-value of 49.48, 45.71, 21.5, 71.13, and 27.31, respectively, and the high values of determination coefficients (R^2^, 0.9802, 0.9786, 0.9556, 0.9861, and 0.9647) for TPC, TFC, ABTS, OH, and DPPH, respectively. The ANOVA results verified that second-order regression models could well match the experimental results and were appropriate for anticipating responses.

TPC was drastically affected by X_2_^2^, followed by X_4_^2^, X_3_^2^, X_2_X_4_, X_4,_ and X_1_^2^. TPC was significantly impacted by the interactive influences: LSR and time, the water content in NADES8 and temperature, and the water content in NADES8 and time. TFC was influenced by X_4_^2^, followed by X_2_^2^, X_1_^2^, X_3_^2^, and X_1_X_4_, and the LSR and time had a mutual effect on the TFC. ABTS was significantly affected by X_3_^2^, followed by X_4_^2^, X_1_^2^, X_1_X_2_, and X_2_^2^. There were mutual interactions: LSR and water content in NADES8, LSR, and time considerably impacted ABTS. OH was substantially influenced by X_3_, followed by X_2_^2^, X_4_^2^, X_1_^2^, and X_3_^2^, and the mutual interaction between LSR and temperature had a significant impact on OH. DPPH was drastically impacted by X_4_^2^, followed by X_1_^2^, X_2_^2^, X_1_, and X_3_, and the interactive effect between LSR and water content in NADES8 exerted a profound influence on DPPH.

**Fig. 6:**
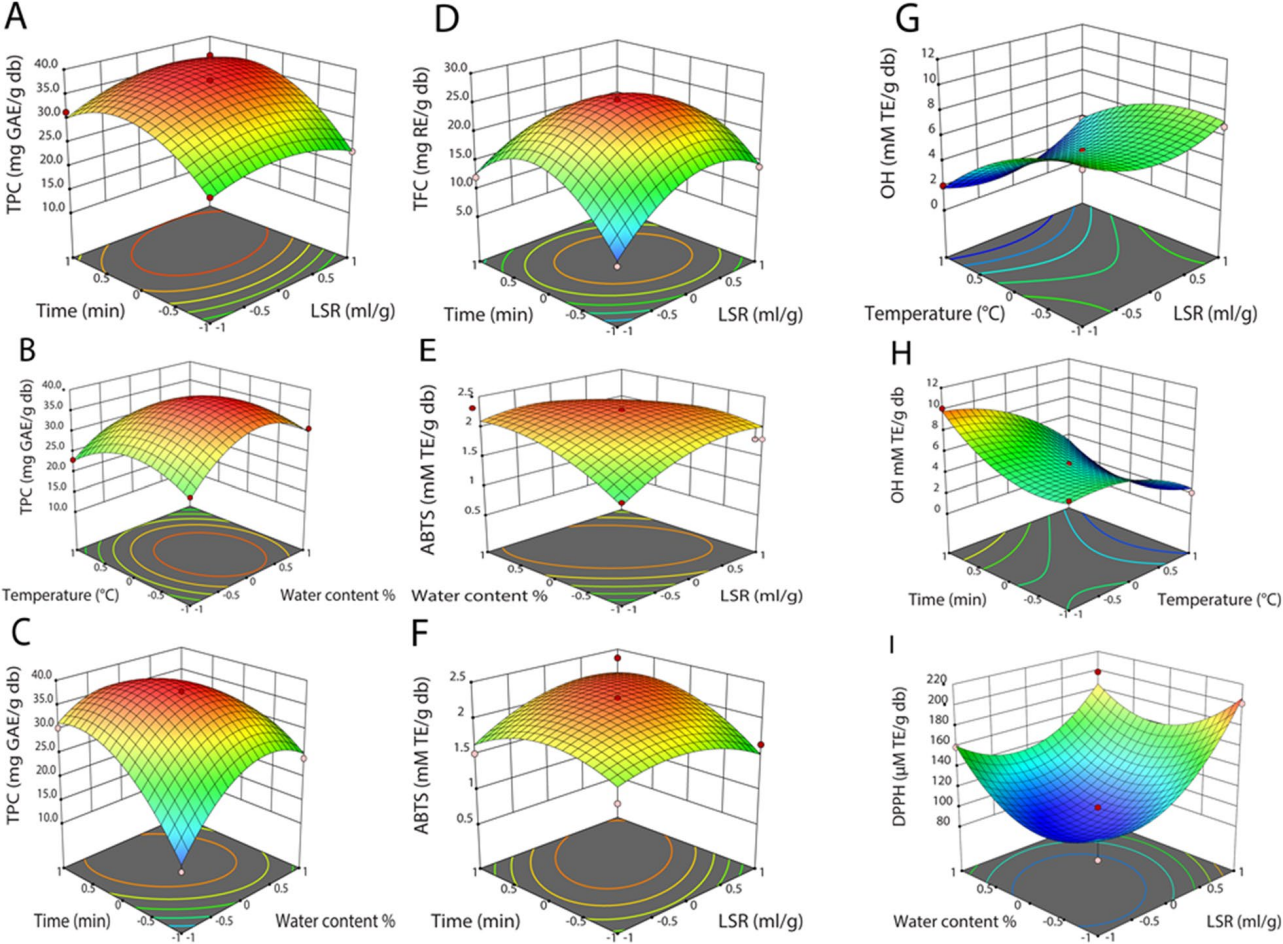
3D response surface plots expressing the significantly interactive effects of independent variables on TPC (**A**-**C**), TFC (**D**), ABTS (**E**–**F**), OH (**G**-**H**), and DPPH (**I**)

Three-dimensional response surface graphics are plotted to clarify the mutual interaction of independent variables, and the mutual interactions significantly affecting TPC, TFC, OH, ABTS, and DPPH are presented in Fig. 6A–I. LSR and time positively affected TPC (Fig. 6A), while the water content in NADES8 and time, the water content in NADES8, and temperature negatively influenced (Fig. 6B, C).

When LSR and time increased, TPC peaked at 36.6 mg GAE/g db. LSR and time negatively impacted TFC (Fig. 6D). TFC significantly rose to 16.8 mg RE/g db with an escalation in LSR and time, followed by a slight decrease. LSR and water content in NADES8 negatively influenced ABTS, while LSR and time had a positive effect. LSR and water content in NADES8 experienced an increase, and ABTS achieved the highest point at 2.2 mM TE/g db, followed by stability. OH was positively affected by LSR and temperature, while OH was negatively impacted by temperature and time. When LSR and temperature increased, there was a substantial decrease in OH. LSR and water content in NADES8 exerted a negative influence on DPPH. As LSR and water content in NADES8 escalated, DPPH significantly grew to 187 µM TE/g db. This finding reached an agreement with Ju-Zhao Liu et al. (2022), who employed NADES to extract natural organic acid from *Hibiscus manihot* L. flower [44]. Therefore, the optimized conditions of the NADES8-based UAE process were 70 ml/g of LSR, 38.9% water content in NADES8, 67.9 °C, and 24.2 min of extraction time to attain maximal TPC, TFC, ABTS, OH, and DPPH at 36.6 mg GAE/g db, 16.8 mg RE/g db, 2.2 mM TE/g db, 8.9 mM TE/g db, and 187 µM TE/g db, respectively.

#### Model validation

The experiments were performed at optimal NADES8-based UAE conditions to validate the confidence of regression models, and the quantified responses are presented in Table 5. Based on the 3D response surface graphics and analyzing the regression of independent variables and responses, the optimal NADES8-based UAE conditions were established at 70 ml/g of LSR, 38.9% water content in NADES8, 67.9 °C, and 24.2 min of extraction time to obtain the predicted values of TPC, TFC, ABTS, OH, and DPPH at 36.6 mg GAE/g db, 16.8 mg RE/g db, 2.2 mM TE/g db, 8.9 mM TE/g db, and 187 µM TE/g db, respectively. The experimental results of TPC, TFC, ABTS, OH, and DPPH obtained at optimal conditions were 38.6 ± 1.8 mg GAE/g db, 16.7 ± 1.2 mg RE/g db, 2.37 ± 0.08 mM TE/g db, 8.32 ± 0.25 mM TE/g db, and 176 ± 7 µM TE/g db, respectively that well fitted with anticipated values with low prediction errors (≤ 7.17%).

**Table 5:** The experimental and predicted values

The variables				Dependent responses	Predicted values	Experimental values	Prediction error %		R^2^_predicted_
LSR ml/g	Water content %	Temperature °C	Time min						
70	38.9	67.9	24.2	TPC (mg GAE/g db)	36.6	38.6 ± 1.8	5.18		0.8859
				TFC (mg RE/g db)	16.8	16.7 ± 1.2	0.60		0.8767
				ABTS (mM TE/g db)	2.2	2.37 ± 0.08	7.17		0.7440
				OH (mM TE/g db)	8.9	8.32 ± 0.25	6.97		0.9201
				DPPH (µM TE/g db)	187	176 ± 7	6.25		0.7965

### The surface morphology of black mulberry fruit powder

**Fig. 7:**
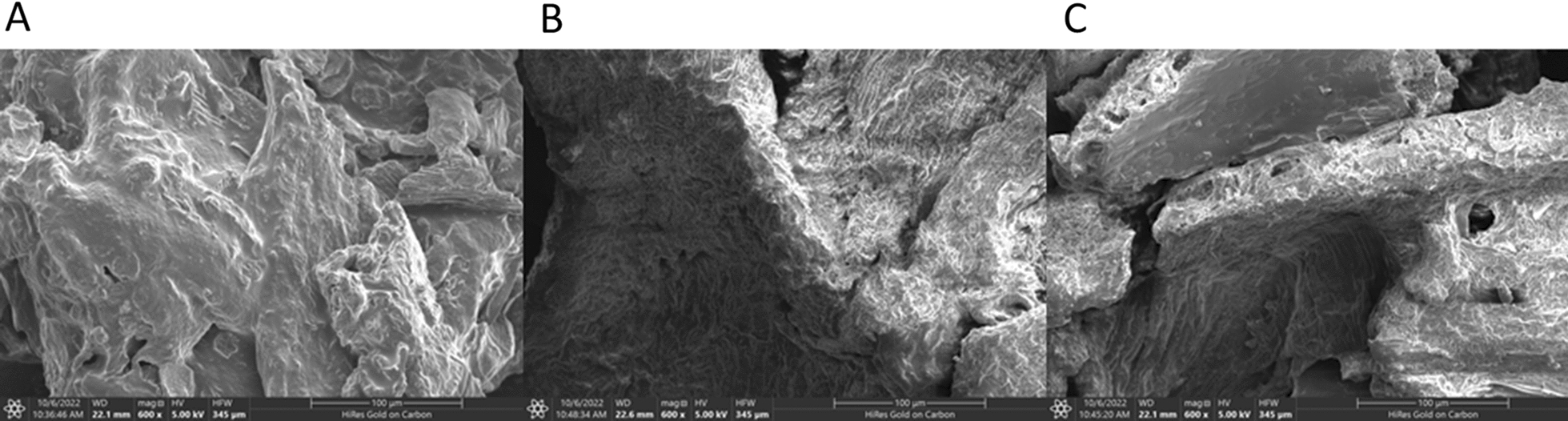
The difference in surface morphology of BMP: **A** surface morphology of BMP without sonication; **B** surface morphology of BMP after ACE60-based UAE; **C** surface morphology of BMP after NADES8-based UAE

The variation in surface morphology of BMP was examined with and without sonication using scanning electron microscopy, and the results are shown in Fig. 7A–C, which was based on a method at 600X magnification. Figure 7A indicated an integral and smooth surface of BMP before sonication, while it was eroded after sonication with ACE60 and NADES8 (Fig. 7B, C). In the case of ACE60-based UAE, the surface of BMP had tiny pores and cracks, possibly due to the acoustic cavitation effect [28]. In the case of NADES8-based UAE, the structural surface of BMP was considerably destroyed and showed large pores, holes, and cracks. It can be attributed to the synergistic effect of the cavitation effect from ultrasound and NADES8. NADES8 with lactic acid as HBD can partially disintegrate cellulose from BMP cell walls, combined with the cavitation effect. This combination can create large pores on the surface of BMP that significantly enhance the permeability of NADES8 into the interior structure of materials. This synergistic effect can improve the extraction of phenolics and flavonoids from BMP more substantially than ACE60-based UAE [28, 38]. This result supports the development of NADES-based UAE as a practical approach for increasing the extraction yield of bioactive compounds from fruits.

## Conclusion

The present study indicated that NADES produced from choline chloride and lactic acid was a suitable solvent for extracting phenolics and flavonoids from BMP and had a higher extraction yield than conventional solvent (ACE60). The optimal NADES8-based UAE conditions were at 70 ml/g of LSR, 38.9% water content, 67.9 °C, and 24.2 min of extraction time to obtain the predicted values of TPC, TFC, ABTS, OH, and DPPH at 36.6 mg GAE/g db, 16.8 mg RE/g db, 2.2 mM TE/g db, 8.9 mM TE/g db, and 187 µM TE/g db, respectively. Scanning electron micrographs showed a more significant change in the surface morphology of BMP after NADES-based UAE than ACE60-based UAE and without sonication. In conclusion, NADES had demonstrated to be a green solvent for the extraction of natural components, and the combination of NADES and UAE was an efficient approach to obtaining the high amount of phenolics and flavonoids from the black mulberry fruit.

## Data Availability

All data generated or analyzed during this study are included in this published article.

## Abbreviations

NADES: Natural deep eutectic solvents
TPC: Total phenolic contents
TFC: Total flavonoid contents
HBA: Hydrogen bond acceptor
HBD: Hydrogen bond donor
UAE: Ultrasonic-assisted extraction
LSR: Liquid-to-solid ratios
ABTS: ABTS+ radical scavenging capacity
DPPH: DPPH radical scavenging capacity
OH: DPPH hydroxyl scavenging capacity
SEM: Scanning electron microscopy
RSM: Response surface methodology
BBD: Box-Behnken design
MAE: Microwave-assisted extraction
ANOVA: Analysis of variance
